# The temporal evolution of family educational priorities and their impact on children’s sports participation: evidence from the HAPC model

**DOI:** 10.3389/fpsyg.2025.1667921

**Published:** 2025-10-07

**Authors:** Yanwei Zong, Aihong Li, Shoudu Wang

**Affiliations:** ^1^College of Physical Education, Yan'an University, Yan'an, China; ^2^College of Accounting (College of Wealth Management), Ningbo University of Finance & Economics, Ningbo, China; ^3^Department of Sports Science, Wenzhou Medical University, Wenzhou, China

**Keywords:** family educational priorities, children’s sports participation, physical activity, generational change, HAPC model

## Abstract

**Introduction:**

This study examines how family educational priorities influence children’s sports participation in China. Drawing on social learning theory and ecological systems theory, it conceptualizes parental emphasis on education as a proximal environmental factor shaping children’s behavioral development through modeling, reinforcement, and value transmission.

**Methods:**

Using nationally representative data from the China Family Panel Studies (CFPS) spanning 2014–2020, the analysis applies a Hierarchical Age-Period-Cohort (HAPC) model to estimate the effects of parental educational emphasis on the frequency and duration of children’s physical activity.

**Results:**

The findings show that stronger parental emphasis on education is positively associated with both the frequency and duration of children’s physical activity. A generational turning point is identified around the 1980 birth cohort, where the impact of parental educational focus shifts from negative to positive. Heterogeneity analysis further indicates that the positive association is more pronounced in urban, two-parent households and among boys.

**Discussion:**

These results highlight the evolving psychological and social mechanisms by which family education priorities shape children’s motivation and participation in physical activity. The findings provide policy-relevant insights for designing equitable, psychologically grounded strategies to promote youth development and reduce disparities in sports participation.

## 1 Introduction

Over the past several decades, China has experienced profound transformations in both family educational values and the broader social environment surrounding physical exercise. Family educational priorities have increasingly influenced the holistic development of children, particularly in relation to their sports participation. Traditionally, both schools and families prioritized academic achievement over physical activity, resulting in limited extracurricular time for exercise and poor physical fitness habits among children. Many parents have underestimated the substantial benefits of physical activity, leading to a lack of family support for physical exercise and the absence of conducive environments for fostering lifelong healthy behaviors. In fact, the World Health Organization (2018) reports that less than 20% of Chinese adolescents meet the recommended physical activity guidelines, signaling a broader public health concern.

To understand how such parental attitudes are internalized by children and reflected in their behavior, we draw on two foundational psychological theories. This neglect can be explained through the lens of ecological systems theory, wherein the family, as part of the microsystem, exerts a direct and sustained influence on children’s behavioral development. When parents de-emphasize physical activity, they not only reduce environmental affordances but also signal its low value, thus shaping children’s health-related behaviors. Furthermore, social learning theory suggests that children acquire behavioral patterns—including sports participation—by observing and imitating parental role models. When parental involvement in physical activity is minimal or discouraged, children are less likely to internalize active lifestyles. Together, these theories emphasize how parenting attitudes and modeling behaviors shape children’s lifestyle choices, especially in formative years (Bandura, 1977; Deci and Ryan, 2000).

**Figure 1:**
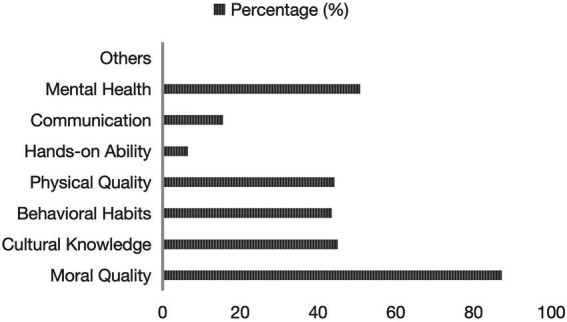
What to focus on in family education. Data source: The *China Children Development Report* (2023).

According to the *China Children Development Report* (2023), the top three priorities identified by Chinese parents in childrearing are moral character (87.2%), mental health (50.7%), and academic achievement (45.0%). In contrast, physical exercise ranks among the most overlooked aspects of parental education (Huo et al., 2023). Despite 44.2% of parents acknowledging the importance of physical fitness, they often neglect or even dismiss the role of regular physical activity. This discrepancy underscores the urgent need to re-evaluate the role of sports within family educational practices (see Figure 1).

From a temporal perspective, parents’ educational expectations are shaped by their own cohort experiences, as different birth cohorts were exposed to varying social and economic conditions. During China’s early reform era, the value of academic credentials was particularly pronounced, given the high returns on education in terms of income and social mobility. As the economy has matured and the criteria for evaluating talent have diversified, the premium placed on academic qualifications has diminished (Liu and Yang, 2022). In parallel, state-led education reforms have promoted more holistic development goals, incorporating physical, moral, and psychological health (Ruan and Tang, 2024). This has ushered in a broader societal emphasis on “quality education,” in which physical and mental health are increasingly viewed as integral components of children’s development. As a result, many parents have shifted from a singular academic focus toward more balanced developmental goals, including physical well-being.

Despite these changing attitudes, children’s actual physical activity has declined in recent years due to urbanization, limited recreational spaces, and mounting academic pressures (Ruan and Tang, 2024). Moreover, gender norms and cultural expectations in Chinese families often shape parental attitudes differently for boys and girls. Traditional beliefs may lead parents to view sports as more appropriate or necessary for boys, potentially contributing to persistent gender disparities in physical activity engagement. Although initiatives such as the “Double Reduction” policy have been implemented to alleviate educational burdens and encourage physical activity, participation levels remain suboptimal, with noticeable disparities by gender and region.

From a developmental psychology perspective, such disparities may reflect sensitive periods in which parental influence is especially potent. During early and middle childhood, parental attitudes can profoundly affect children’s self-perceptions of competence and autonomy—key components of self-determination theory (Deci and Ryan, 2000), which underscores the role of intrinsic motivation in sustaining behaviors like sports participation.

This raises a set of critical and underexplored questions: How does children’s sports participation evolve across age, historical periods, and generational cohorts? How does the influence of family educational priorities on children’s physical activity vary by gender and regional context over time? Do these patterns exhibit convergence or divergence across demographic groups? Existing literature has yet to offer comprehensive answers to these questions.

To address this gap, this study draws on nationally representative data from the China Family Panel Studies (2014–2020) and employs the Hierarchical Age-Period-Cohort (HAPC) model to systematically examine how family educational priorities shape children’s sports participation. By incorporating life course theory and a temporal analytical framework, this paper provides a nuanced understanding of how family attitudes toward education influence children’s physical activity across generations and demographic groups.

This study makes three main contributions. First, it adopts a novel generational perspective to explore how the relationship between family educational expectations and children’s sports behavior evolves over time. Second, it enriches the empirical literature by examining the heterogeneous effects of family educational priorities using a robust HAPC approach, addressing potential endogeneity and conducting extensive robustness checks. Third, it generates practical policy recommendations to improve children’s sports participation through informed and culturally sensitive family education strategies.

## 2 Literature review and research hypothesis

Family Education Focus refers to parents’ investments and expectations in their children’s education, encompassing material resource investment (economic capital), utilization of social resources (social capital), and the transmission of cultural capital. The family capital theory provides a systematic framework for understanding family education focus. By analyzing how a family’s economic, social, and cultural capital influences educational investments and expectations, we can gain a better understanding of the various dimensions of family education focus. Family capital theory emphasizes the significant impact of a family’s economic, social, and cultural capital on individual education and development (Coleman, 1988; Lareau, 2018). Economic capital refers to the material resources of the family, social capital pertains to the family’s social networks and relationships, and cultural capital includes the family’s educational background, cultural literacy, and educational investments. Existing research indicates that family capital plays a crucial role in children’s education and sports participation (Xue, 2019; McMillan et al., 2016; Dai and Li, 2022). High-education-level families maintain their social status through educational resource investment and the transmission of cultural capital. Family education focus, such as parents’ educational expectations and residential moves for better school districts, is a specific manifestation of family cultural capital, significantly affecting children’s academic and sports participation (Hayoz et al., 2019). As part of holistic development, sports participation has become an essential component of family cultural capital (Tan et al., 2023).

### 2.1 Mechanisms of the influence of family education focus on children’s sports participation

Family education focus can influence children’s sports involvement through multiple pathways, including material resources, parental expectations, and behavioral modeling. Building on family capital theory, these influences extend beyond structural factors to include psychological mechanisms rooted in developmental and motivational theories.

The influence of family education focus on children’s sports participation can first be understood through the lens of family capital theory. According to this framework, families deploy economic, social, and cultural capital to support their children’s development (Coleman, 1988; Lareau, 2018). Economic capital provides material resources (e.g., sports equipment, access to facilities); social capital entails supportive networks that may enable participation (e.g., peer or parental groups); and cultural capital reflects values and aspirations—including the extent to which physical activity is seen as integral to educational success. Prior studies have shown that families with higher educational backgrounds tend to invest more in structured activities—including sports—as part of a broader strategy for upward mobility and status transmission (Xue, 2019; Tan et al., 2023). Therefore, family education focus functions not only through academic expectation but also through the structural provision of opportunities and values that support active lifestyles.

Beyond structural factors, psychological mechanisms also mediate the effect of family education focus. Drawing on social learning theory (Bandura, 1977), children internalize behaviors by observing parental role models. Parents who participate in or value sports provide visible behavioral cues that shape children’s own habits. Simultaneously, ecological systems theory (Bronfenbrenner, 1986) emphasizes the role of the family as a microsystem that constructs daily environments—through routines, scheduling, and norms—that either enable or suppress physical activity. Finally, self-determination theory (Deci and Ryan, 2000) explains how parenting practices that support children’s autonomy, competence, and relatedness can foster intrinsic motivation for sustained participation. When family education focus is coupled with autonomy-supportive parenting and emotional involvement, children are more likely to develop a self-driven engagement in physical activity.

Taken together, these theories suggest that family education focus affects sports participation not only by allocating resources, but also by transmitting values, structuring environments, and shaping motivational climates.

*Hypothesis 1:* There is a positive correlation between family education focus and children's sports participation.

### 2.2 Temporal changes in the impact of family education focus on children’s sports participation

The evolution of family education in China from a focus on academic achievements to quality education reflects a shift in educational philosophy and goals. With societal development and educational system reforms, the importance of family education has become increasingly prominent. Family education now emphasizes not only academic performance but also the cultivation of children’s comprehensive qualities and values (Gao, 2016; Zhou et al., 2024). The concept of quality education highlights the development of students’ innovative abilities, practical skills, and overall qualities, significantly influencing Chinese family education (Owen et al., 2022; Su et al., 2024). Moreover, the shift from academic education to a focus on physical education reflects China’s emphasis on holistic development. Numerous studies underline the importance of promoting physical education (Wang et al., 2023; Židek, 2024). Participation in sports not only enhances children’s physical fitness but also improves concentration, communication skills, teamwork, and stress resilience (Schlesinger and Nagel, 2013; Mutz and Albrecht, 2017). Chinese parents are increasingly recognizing the importance of their children’s physical and mental health and are beginning to value and support their participation in sports (Liu et al., 2024).

#### 2.2.1 Changes with age

As children grow, the influence of family education on their sports participation exhibits distinct phased characteristics. There are significant differences in how children of different ages engage in sports and in the focal points of parental attention. Young children rely more on direct guidance and accompaniment from parents, while adolescents display greater autonomy, though they remain deeply influenced by the family environment and parental attitudes. For example, Onywera et al. (2012) found that rural children had significantly higher daily step counts than urban children, indicating that at different age stages, the level of parental focus and guidance on sports significantly affects children’s participation. Wex et al. (2023) noted that young children in urban environments have less physical activity primarily due to parental safety concerns, but as children grow older, parental focus shifts to balancing safety with increased opportunities for physical activity. Ludwig et al. (2022) emphasized that the family environment during early childhood is crucial for forming early exercise habits, with parental involvement and encouragement being particularly important at this stage.

#### 2.2.2 Changes over time

The impact of family education concepts and social-cultural backgrounds on children’s sports participation varies across different historical periods. The rapid development of modern society and technological advancements have significantly altered lifestyles, especially in urbanization processes, profoundly affecting children’s exercise habits. However, in the early 21st century, with technological advancements, parents increasingly focused on balancing children’s screen time with physical activity. During times of economic prosperity, parents emphasized comprehensive development for their children, including physical activities. Ezenwugo (2020) showed significant differences in how family education influenced children’s sports participation across different historical periods. Earlier families emphasized outdoor activities and physical exercise, while modern families prioritize organizing physical activities in safe environments rich in educational resources. Ludwig et al. (2022) pointed out that rapid urbanization and lifestyle changes significantly impacted children’s physical activity levels, with the proliferation of modern technology reducing outdoor playtime. Wex et al. (2023) found a recent decline in active school transportation (AST) among children in urban areas, primarily due to parental safety concerns and the lack of relevant infrastructure. This highlights how changing societal conditions and technological advancements influence family education practices and children’s sports participation over time.

#### 2.2.3 Changes across birth cohorts

The educational methods and focal points of families from different birth cohorts have continuously evolved, reflecting the impact of changing times on children’s sports participation. Early research primarily focused on physical health and the development of motor skills. In recent years, however, psychological health and the development of comprehensive qualities have gradually become key concerns for parents. Stalsberg and Pedersen (2010) studied the impact of socioeconomic status on physical activity and found that the emphasis on and methods of engaging in sports varied among families from different birth cohorts. Ludwig et al. (2022) studied countries in sub-Saharan Africa and found significant differences in the physical activity levels of children from different birth cohorts during urbanization processes. These findings underscore the evolving nature of family education focus and its impact on children’s sports participation across different generations.

*Hypothesis 2:* The impact of family education focus on children's sports participation varies across different periods and cohorts.

## 3 Research design

### 3.1 Data source and sample selection

This study utilizes data from the China Family Panel Studies (CFPS) collected between 2014 and 2020. The CFPS aims to track and collect data at the individual, family, and community levels, reflecting changes in China’s society, economy, population, education, and health. It provides a robust data foundation for academic research and public policy analysis. The CFPS focuses on both economic and non-economic well-being of Chinese residents, covering a wide range of research topics including economic activities, educational outcomes, family relationships and dynamics, population migration, and health. As a nationwide, large-scale, multidisciplinary social tracking survey project, CFPS offers comprehensive insights into various aspects of Chinese life. Given that this study examines the impact of family education focus on children’s sports participation, families with children were selected as the research subjects. The sample was further refined based on the relevance to the study’s objectives.

### 3.2 Variable selection

#### 3.2.1 Dependent variable

The dependent variable in this study is children’s sports participation. The CFPS data includes information on the frequency of children’s sports participation per month and the duration of children’s sports participation per month (measured in hours). This data primarily pertains to children aged 10–16. This age range was chosen because the CFPS dataset only consistently reports children’s sports behavior variables for those aged 10 and above, and by age 16, children are typically transitioning into late adolescence, where independent decision-making may reduce parental influence. Although the WHO defines children as those aged 0–18, this study focuses on the 10–16 group due to data availability and the theoretical relevance of parental influence during early to middle adolescence. Therefore, this study focuses on samples from valid questionnaires within this age range.

#### 3.2.2 Key independent variables

The key independent variable in this study is family education focus, along with the age, period, and birth cohort of the household head. We use the CFPS survey question “What is the minimum level of education you hope your child will complete?” as the primary proxy for family education focus. The possible responses are: “2. Elementary School; 3. Middle School; 4. High School; 5. Junior College; 6. Undergraduate; 7. Master’s; 8. Doctorate.” Option “1” (No schooling) was not included in the analysis because it was not presented as a valid response option in the questionnaire module related to parental aspirations. For robustness checks, the variable “consideration of studying abroad” is used for benchmark regression verification, with “1” indicating consideration and “0” indicating no consideration.

The reasons for selecting the age, period, and birth cohort of the household head as key independent variables are grounded in the household head’s role as the primary decision-maker in family education decisions. In the CFPS data, the household head is identified as the primary decision-maker in the family, making it logical to use their age, period, and birth cohort characteristics as key independent variables. The sample’s age range is 26–84 years. The periods are defined by the survey years: 2014–2015, 2016–2017, 2018–2019, and 2020–2021, resulting in four periods. Birth cohorts are divided into five-year intervals, ranging from 1930–1934 to 1990–1994, yielding a total of 13 birth cohort groups.

#### 3.2.3 Instrumental variable

There is a bidirectional causality and endogeneity issue between family education focus and children’s sports participation. The time children spend on sports and the outcomes they achieve may influence parents’ choices regarding their children’s future educational direction, thereby affecting family education focus. Additionally, both family education focus and children’s sports participation are influenced by unmeasured variables such as cultural background and personality traits, which serve as omitted variables in the econometric estimation.

To address the endogeneity issue, this study selects the number of books in the household and the average educational expectations of parents in the same community as instrumental variables. The number of books in the household reflects the parents’ emphasis on education, with a larger collection indicating greater attention to their children’s education, thus positively correlating with family education focus. The average educational expectations of parents in the same community reflect the educational cultural atmosphere of the community, which influences parents’ educational attitudes and behaviors, thereby affecting family education focus. The number of books in the household is highly correlated with family education focus but does not directly influence children’s sports participation, only indirectly through family education focus. Similarly, the average educational expectations of parents in the same community indirectly influence children’s sports participation by affecting parents’ education focus and do not have a direct impact. Therefore, these two instrumental variables meet the exogeneity condition and can effectively resolve the endogeneity issue.

#### 3.2.4 Control variables

Based on the availability and continuity of multi-year survey data, the control variables in this study include household head characteristics, child characteristics, family characteristics, and regional variables. Specifically, the symbols, meanings, and measurements of the main variables are detailed in Table 1, and the descriptive statistics of the main variables are presented in Table 2.

**Table 1:** Symbolic meaning and measurement of the main variables.

Variable Classification	Variables	Definition
Dependent variables	kidsport_fr	Children’s monthly sports participation frequency
	kidsport_hr	Children’s monthly sports participation hours
Core independent variables	child_education_level	Parents’ expected minimum education level for child
	consider_abroad_education	Whether parents consider sending child abroad for education (1 = yes, 0 = no)
Household level	household_age	Age of household head
	household_gender	Gender of household head
	household_eduy	Years of education of household head
Child level	kidage	Age of child
	kidgender	Gender of child
	kidbmi	Child’s BMI
Family level	familysize	Number of family members
	econ_status	Subjective economic status
	lnresivalue_tho	ln(property value + 1)
Regional level	urban	1 if residing in urban area, 0 if in rural area
	provcd	Province dummy variables
Period		Survey conducted every two years, values based on survey year
cohortt_group		Cohort groups based on household head’s birth year, with each cohort spanning 5 years

**Table 2:** Descriptive statistics of the main variables.

Variables	*N*	Mean	SD	Min	Max
kidsport_fr	7,234	3.465	2.977	0	26
kidsport_hr	7,234	8.692	21.288	0	300
child_education_level	7,234	6.209	1.192	2	9
consider_abroad_education	7,234	0.172	0.377	0	1
household_age	7,234	48.051	11.762	26	84
household_gender	7,234	0.720	0.449	0	1
household_eduy	7,234	7.370	4.210	0	22
kidage	7,234	12.430	1.712	9	16
kidgender	7,234	0.531	0.499	0	1
kidbmi	7,234	18.428	3.728	12	40
familysize	7,234	5.045	1.840	1	17
econ_status	7,234	3.019	1.016	1	5
lnresivalue_tho	7,234	4.660	1.842	0	9.21
urban	7,234	0.419	0.493	0	1
provcd	7,234	40.287	15.062	11	65

### 3.3 Research methodology

This study focuses on the changing trends in the impact of family education focus on children’s sports participation across age, period, and cohort. To elucidate the relationships among these variables, this study employs the Hierarchical Age-Period-Cohort (HAPC) model to examine the independent effects of age, period, and cohort. Traditional APC models may face estimation instability and interpretational difficulties due to the complete collinearity problem and model identification issues. In contrast, the HAPC model addresses these challenges by introducing random effects, allowing for better handling of hierarchical data structures, partially mitigating collinearity issues, and providing more flexible and accurate estimates (Yang and Land, 2013). Traditional APC models mainly rely on fixed effects estimation, which cannot effectively capture the hierarchical structure and random variation in the data. The HAPC model, by incorporating random effects for periods and cohorts, better handles the hierarchical relationships in the data, capturing variability within these levels. This approach enhances the precision and interpretability of the model estimates. According to the research hypothesis, the mathematical expression of the HAPC model for the impact of family education focus on children’s sports participation is as follows (see Equations 1–3):

1. First-level model: Estimating individual-level effects

(1) $$\begin{array}{l} Y_{\mathrm{ijk}}=\beta_{0}+\beta_{1}{\text{HouseholdLevel}}_{\mathrm{ijk}}+\beta_{2}{\text{ChildLevel}}_{\mathrm{ijk}}+\\ \beta_{3}{\text{FamilyLevel}}_{\mathrm{ijk}}+\ldots +u_{j}+v_{k}+\epsilon_{\mathrm{ijk}} \end{array}$$

Where $Y_{\mathrm{ijk}}$ is the dependent variable value for the *i*-th individual in the *j*-th period and *k*-th cohort. $\beta_{0}$ is the intercept term, and $\beta_{1},\beta_{2},\beta_{3}$ are the coefficients for the individual-level independent variables (including household level, child level, and family level variables). ${\text{HouseholdLevel}}_{\mathrm{ijk}}$ represents the household-level independent variables, ${\text{ChildLevel}}_{\mathrm{ijk}}$ represents the child-level independent variables, and ${\text{FamilyLevel}}_{\mathrm{ijk}}$ represents the family-level independent variables. $u_{j}$ is the period random effect, $v_{k}$ is the cohort random effect, and $\epsilon_{\mathrm{ijk}}$ is the individual-level error term.

1. Second-level model: Estimating the effects of period and cohort variables

(2) $$\beta_{0\mathrm{jk}}=\gamma_{0}+\gamma_{1}{\text{Period}}_{j}+\gamma_{2}{\text{Cohort}}_{k}+\gamma_{3}{\text{Region}}_{\mathrm{jk}}+u_{j}+v_{k}$$

Where: $\beta_{0\mathrm{jk}}$ is the intercept term for the *j*-th period and *k*-th cohort. $\gamma_{0}$ is the overall average intercept. $\gamma_{1}{\text{Period}}_{j}$ is the period effect. $\gamma_{2}{\text{Cohort}}_{k}$ is the cohort effect. $\gamma_{3}{\text{Region}}_{\mathrm{jk}}$ is the regional level effect. $u_{j}$ is the period random effect. $v_{k}$ is the cohort random effect.

Combining the above two-level models, we obtain the complete HAPC model:


(3) $$\begin{array}{l} Y_{\mathrm{ijk}}=\gamma_{0}+\gamma_{1}{\text{Period}}_{j}+\gamma_{2}{\text{Cohort}}_{k}+\gamma_{3}{\text{Region}}_{\mathrm{jk}}+\\ \beta_{1}{\text{HouseholdLevel}}_{\mathrm{ijk}}+\beta_{2}{\text{ChildLevel}}_{\mathrm{ijk}}+\\ \beta_{3}{\text{FamilyLevel}}_{\mathrm{ijk}}+u_{j}+v_{k}+\epsilon_{\mathrm{ijk}} \end{array}$$

Where: $\gamma_{0}$ is the overall average intercept. $\gamma_{1}{\text{Period}}_{j}$ is the period effect. $\gamma_{2}{\text{Cohort}}_{k}$ is the cohort effect. $\gamma_{3}{\text{Region}}_{\mathrm{jk}}$ is the regional level effect. $\beta_{1},\beta_{2},\beta_{3}$ are the coefficients of the individual-level independent variables (including household level, child level, and family level). ${\text{HouseholdLevel}}_{\mathrm{ijk}},{\text{ChildLevel}}_{\mathrm{ijk}},{\text{FamilyLevel}}_{\mathrm{ijk}}$ are the individual-level independent variables. $u_{j}$ is the period random effect. $v_{k}$ is the cohort random effect. $\epsilon_{\mathrm{ijk}}$ is the individual-level error term.

## 4 Analysis of estimation results

### 4.1 Benchmark results

Table 3 presents the benchmark models of the HAPC analysis conducted in this study, focusing on the impact of family education focus on children’s sports participation frequency and duration after controlling for individual, family, and regional characteristics. The benchmark regression reports both fixed and random effects. The fixed effects are implemented through a stepwise regression approach, progressively adding control variables at the household and child levels, family level, and regional level to enhance the robustness of the overall estimates. The fixed effects results of the benchmark regression reveal that family education focus significantly positively impacts children’s sports participation frequency and duration at the 1% level. Specifically, for each unit increase in parents’ educational expectations for their children, the score of children’s sports participation frequency increases by approximately 0.084 to 0.113 units. The education level of the household head also significantly positively affects children’s sports participation. Regarding the impact of children’s age and the square of children’s age on sports participation frequency, there is an inverted U-shaped trend. It is also evident that boys are more influenced by family education focus in sports participation than girls. Additionally, the larger the family size, the smaller the impact of family education focus on children’s sports participation; urban families have a significantly greater impact compared to rural families. These results provide a solid foundation for further analysis.

**Table 3:** Benchmark regression result.

Variables	kidsport_fr			kidsport_hr		
	(1)	(2)	(3)	(4)	(5)	(6)
Fixed effect						
child_education_level	0.113***	0.107***	0.084***	0.755***	0.711***	0.606***
	(0.031)	(0.031)	(0.031)	(0.208)	(0.209)	(0.209)
household_age	0.034	0.033	0.024	0.055**	0.067	0.049
	(0.023)	(0.023)	(0.023)	(0.028)	(0.158)	(0.158)
household_gender	−0.137*	−0.122*	−0.104	−0.463	−0.321	−0.282
	(0.073)	(0.073)	(0.074)	(0.491)	(0.493)	(0.501)
household_eduy	0.055***	0.050***	0.042***	0.228***	0.190***	0.122**
	(0.008)	(0.008)	(0.009)	(0.055)	(0.056)	(0.061)
kidage	1.018***	0.989***	0.997***	2.307	2.124	2.174
	(0.316)	(0.316)	(0.315)	(2.135)	(2.134)	(2.123)
kidage_sq	−0.039***	−0.038***	−0.038***	−0.069	−0.063	−0.065
	(0.013)	(0.013)	(0.013)	(0.085)	(0.085)	(0.085)
kidgender	0.353***	0.337***	0.367***	2.439***	2.328***	2.360***
	(0.065)	(0.065)	(0.065)	(0.438)	(0.439)	(0.439)
kidbmi	0.004	0.003	−0.003	0.101*	0.095	0.102*
	(0.009)	(0.009)	(0.009)	(0.059)	(0.059)	(0.060)
familysize		−0.047**	−0.010		−0.355***	−0.277**
		(0.019)	(0.020)		(0.127)	(0.133)
lnresivalue_tho		0.035*	0.038**		0.261**	0.115
		(0.018)	(0.019)		(0.122)	(0.125)
econ_status		0.050	0.055*		0.020	0.086
		(0.032)	(0.033)		(0.218)	(0.220)
urban			0.253***			2.111***
			(0.073)			(0.495)
cohort_group	0.026	0.025	0.016	-	0.008	−0.001
	(0.023)	(0.023)	(0.023)	-	(0.157)	(0.157)
Province	control	control	control	control	control	control
Stochastic effect						
cohort_group						
1930–1934	−0.621***	−0.620***	−0.653***	−6.123***	−6.089***	−6.232***
	(0.002)	(0.002)	(0.002)	(0.001)	(0.001)	(0.000)
1935–1939	−0.269***	−0.269***	−0.285***	−2.891***	−2.872***	−2.950***
	(0.010)	(0.010)	(0.010)	(0.101)	(0.101)	(0.102)
1940–1944	−0.516***	−0.519***	−0.529***	−5.022***	−5.035***	−5.076***
	(0.007)	(0.007)	(0.007)	(0.064)	(0.064)	(0.065)
1945–1949	−0.280***	−0.281***	−0.290***	−2.755***	−2.755***	−2.791***
	(0.010)	(0.010)	(0.011)	(0.102)	(0.102)	(0.103)
1950–1954	−0.173***	−0.176***	−0.175***	−1.751***	−1.767***	−1.762***
	(0.011)	(0.011)	(0.012)	(0.114)	(0.114)	(0.115)
1955–1959	−0.050***	−0.051***	−0.049***	−0.460***	−0.470***	−0.457***
	(0.013)	(0.013)	(0.013)	(0.125)	(0.125)	(0.126)
1960–1964	0.103***	0.103***	0.107***	0.937***	0.934***	0.951***
	(0.013)	(0.013)	(0.014)	(0.134)	(0.134)	(0.135)
1965–1969	−0.318***	−0.320***	−0.332***	−3.439***	−3.433***	−3.495***
	(0.009)	(0.009)	(0.009)	(0.092)	(0.093)	(0.094)
1970–1974	−0.260***	−0.261***	−0.271***	−2.819***	−2.816***	−2.864***
	(0.010)	(0.010)	(0.010)	(0.101)	(0.101)	(0.102)
1975–1979	−0.157***	−0.158***	−0.164***	−1.794***	−1.794***	−1.822***
	(0.011)	(0.011)	(0.011)	(0.113)	(0.113)	(0.114)
1980–1984	0.214***	0.214***	0.225***	2.105***	2.094***	2.141***
	(0.014)	(0.014)	(0.014)	(0.140)	(0.140)	(0.141)
1985–1989	0.748***	0.751***	0.775***	7.363***	7.362***	7.461***
	(0.014)	(0.015)	(0.015)	(0.148)	(0.149)	(0.149)
1990–1994	1.464***	1.473***	1.506***	14.586***	14.617***	14.756***
	(0.011)	(0.011)	(0.011)	(0.114)	(0.115)	(0.115)
_all: Identity var.(R.cohort_group)	0.000	0.000	0.000	0.752	0.645	0.665
	(0.000)	(0.000)	(0.000)	(0.761)	(0.690)	(0.719)
period: Identity var.(_cons)	1.180	1.196	1.242	118.248	119.0153	120.826
	(0.842)	(0.854)	(0.886)	(86.798)	(84.535)	(85.818)
LR test vs. linear model: chi2(2)	559.62***	567.18***	579.09***	1726.80***	1200.88***	1214.81***
Constant	−56.257	−54.500	−36.678	−20.059	−33.003	−13.908
	(46.222)	(46.203)	(46.101)	(14.384)	(316.716)	(316.176)
Observations	7,234	7,234	7,234	7,234	7,234	7,234

Standard errors in parentheses, ***, ** and * represent significant at the 1, 5 and 10% levels, respectively.

In this study, the household head’s age is divided into cohorts every 5 years, and periods are categorized based on the survey years. It was found that the period effects are minimal; hence, the benchmark regression’s random effects primarily report each cohort’s effects, standard errors, and significance, as well as the overall cohort effects, period effects, and the fit of the HAPC model. To visually present the cohort effects, trend estimation graphs are used.

**Figure 2:**
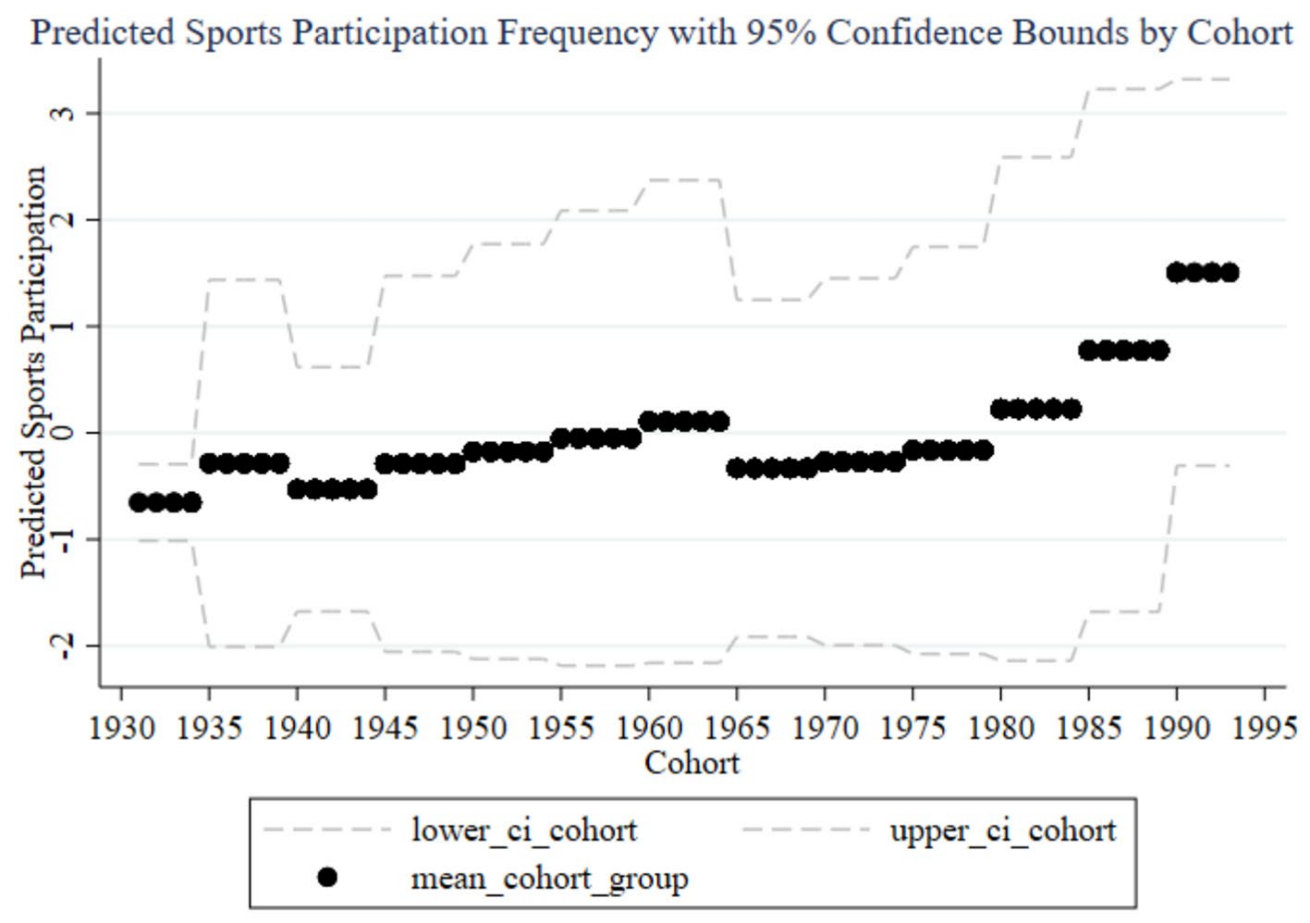
Trends in the impact of family education focus on children’s sports participation frequency across cohorts.

Figure 2 shows the trend of the impact of family education focus on children’s sports participation frequency across different birth cohorts. The graph includes predicted participation frequency and 95% confidence intervals. The black dots represent the average participation frequency for each cohort group, while the dashed lines indicate the 95% confidence intervals. From 1930 to 1995, the impact of parents’ education focus on children’s sports participation frequency shows an overall upward trend, particularly increasing significantly in the cohorts of parents born in the 1980s and 1990s. Observing the trends from early, mid, and late cohorts, it is evident that the impact of family education focus on children’s sports participation frequency is generally low with minimal variation in the early cohorts (1930–1960), and the wide confidence intervals suggest high estimation uncertainty. In the mid cohorts (1960–1980), the impact starts to gradually increase, and the confidence intervals begin to narrow, indicating improved estimation accuracy. In the late cohorts (1980–1995), the impact significantly rises, reaching a peak in the 1985–1989 and 1990–1994 cohorts, with the narrow confidence intervals suggesting high estimation precision. Therefore, family education focus has a significant positive impact on children’s sports participation frequency in the late cohorts. Parents born in the 1980s and 1990s particularly exhibit increased influence on their children’s sports participation frequency, which may be related to changing family education concepts. Over time, the confidence intervals for different cohorts gradually narrow, indicating more precise estimates, likely due to larger sample sizes and improved data quality in the late cohorts. In the early cohorts, the impact of family education focus on children’s sports participation frequency is small and unstable. As time progresses, the importance of family education focus increases, producing a significant positive impact on children’s sports participation frequency in the later cohorts.

**Figure 3:**
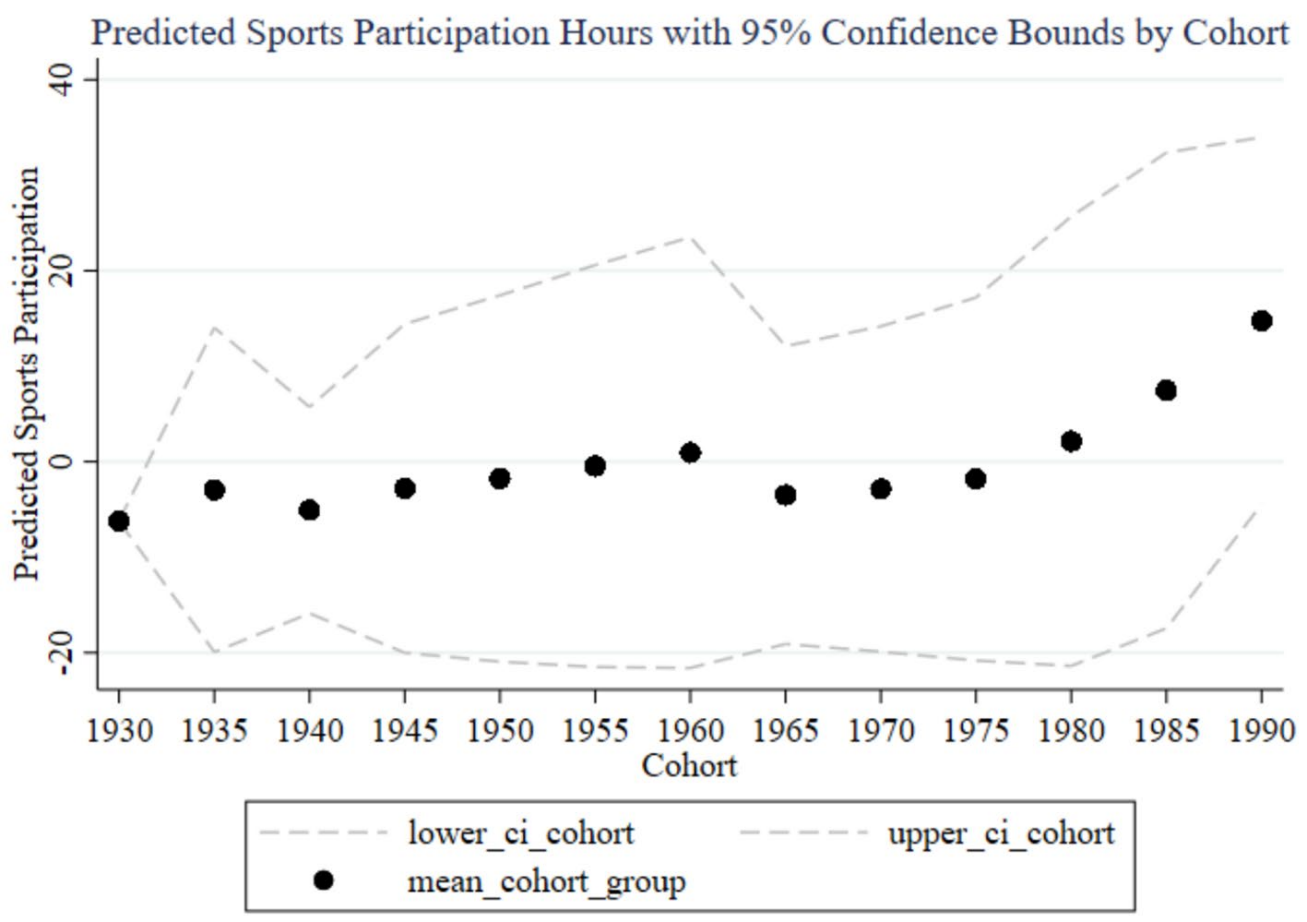
Trends in the impact of family education focus on children’s sports participation duration across cohorts. “lower_ci_cohort” correspond to the lower 95% confidence interval “upper_ci_cohort” correspond to the upper 95% confidence interval. The same labeling logic applies to subsequent figures in the manuscript.

Figure 3 illustrates the trend of the impact of family education focus on children’s sports participation duration across different birth cohorts, including predicted participation duration and 95% confidence intervals. The black dots represent the average participation duration for each cohort group, while the dashed lines indicate the 95% confidence intervals. From 1930 to 1990, the influence of parents’ education focus on children’s sports participation duration shows an overall upward trend, with a significant increase in the cohorts born in the 1980s and 1990s. Observing the trends from early, mid, and late cohorts, it is evident that the impact of parents’ education focus on children’s sports participation duration is low with minimal variation in the early cohorts (1930–1960), and the wide confidence intervals suggest high estimation uncertainty. In the mid cohorts (1960–1980), the impact starts to gradually increase, and the confidence intervals begin to narrow, indicating improved estimation accuracy. In the late cohorts (1980–1990), the impact significantly rises, reaching a peak in the 1985–1989 and 1990–1994 cohorts, with the narrow confidence intervals suggesting high estimation precision. Therefore, family education focus has a significant positive impact on children’s sports participation duration in the late cohorts.

From the random effects in Table 3 and the results reported in Figures 2, 3, it is evident that each cohort has a significant impact. Interestingly, using 1980 as a boundary, the cohort effects before this year are predominantly significantly negative (except for the 1960–1964 cohort), while the cohort effects after 1980 are significantly positive. This indicates that parents born before 1980 generally believed that sports participation negatively affected academic performance, so the higher their educational expectations, the less their children participated in sports. In contrast, parents born after 1980 placed more emphasis on holistic development and believed that children’s sports participation could enhance academic performance. This aligns with the changing trends in educational concepts in China.

### 4.2 Robustness checks

#### 4.2.1 Robustness check for addressing endogeneity

This study employs the two-stage least squares regression (2SLS) method for instrumental variable testing. Table 4 reports the estimation results. After incorporating the instrumental variables—the number of books in the household and the average educational expectations in the residential community—the impact of family education focus on children’s sports participation frequency and duration remains significantly positive at the 1% level. In the study, the logarithm of property value was taken to address heteroscedasticity, so the Anderson LM statistic was used for the under-identification test. The chi-square *p*-value is significant at the 1% level, rejecting the null hypothesis of under-identification, indicating that the instrumental variables are identifiable. The Cragg-Donald Wald F statistic for weak instrument identification is greater than the 10% maximal IV size value of 19.93, suggesting that the selected instrumental variables are not weak instruments. The Sargan statistic was used for the over-identification test, and the chi-square p-value is not significant, failing to reject the null hypothesis that all instrumental variables are exogenous. This indicates that all instrumental variables are exogenous. From the analysis, it is evident that both instrumental variables are neither weak nor endogenous. Thus, the results from the instrumental variable test for endogeneity are consistent with the benchmark regression, confirming that family education focus has a significantly positive impact on children’s sports participation frequency and duration after addressing endogeneity.

**Table 4:** Robustness test: endogeneity test regression results.

Variables	IV_results	
	(1)	(2)
child_education_level	0.352***	2.026***
	(0.057)	(0.415)
household_age	0.380***	3.244***
	(0.022)	(0.201)
household_gender	−0.081	0.420
	(0.096)	(0.798)
household_eduy	0.045***	0.278***
	(0.011)	(0.086)
kidage	1.061***	4.062
	(0.406)	(3.213)
kidage_sq	−0.042***	−0.131
	(0.016)	(0.130)
kidgender	0.390***	3.258***
	(0.084)	(0.663)
kidbmi	0.003	0.128
	(0.011)	(0.097)
familysize	−0.002	−0.052
	(0.026)	(0.175)
lnresivalue_tho	−0.016	−0.103
	(0.024)	(0.227)
econ_status	0.043	−0.054
	(0.040)	(0.312)
urban	0.153*	2.359***
	(0.092)	(0.779)
cohort_group	0.372***	3.167***
	(0.021)	(0.193)
Constant	−757.049***	−6,426.925***
	(42.199)	(389.964)
Observations	4,889	4,889
R-squared	0.104	0.108
Under identification test	898.594	898.594
	(0.000)	(0.000)
Weak identification test	1996.443	1996.443
	(19.930)	(19.930)
Sargan statistic	2.120	0.989
	(0.145)	(0.320)

***, **, and * indicate significance at the 1, 5, and 10% levels, respectively. The values in parentheses next to the main regression results are standard errors. For the under-identification test, the value in parentheses is the chi-square *p*-value of the Anderson LM statistic. For the weak identification test, the value in parentheses is the Cragg-Donald Wald F statistic relative to the 10% maximal IV size reference value. For the Sargan statistic, the value in parentheses is the chi-square *p*-value.

#### 4.2.2 Robustness check by changing the independent variable

To further verify the robustness of the benchmark regression, this study conducts a robustness check by changing the independent variable. The study uses the CFPS questionnaire item “Have you considered sending your child to study abroad?” as a proxy variable for family education focus. A dummy variable is generated based on the response to this question, with a value of 1 indicating consideration and 0 indicating no consideration. The HAPC model is then estimated using this variable. The results remain significantly positive at the 1% level, consistent with the benchmark regression (see Table 5).

**Table 5:** Robustness check: changing the independent variable.

Variables	kidsport_fr			kidsport_hr		
	(1)	(2)	(3)	(4)	(5)	(6)
Fixed effect						
consider_abroad_education	0.218**	0.206**	0.194**	1.447**	1.382**	1.095*
	(0.086)	(0.086)	(0.086)	(0.579)	(0.579)	(0.582)
household_age	0.033	0.033	0.024	0.078	0.067	0.046
	(0.023)	(0.023)	(0.023)	(0.158)	(0.158)	(0.158)
household_gender	−0.150**	−0.133*	−0.110	−0.549	−0.393	−0.328
	(0.073)	(0.073)	(0.074)	(0.490)	(0.493)	(0.501)
household_eduy	0.060***	0.055***	0.046***	0.263***	0.222***	0.146**
	(0.008)	(0.008)	(0.009)	(0.054)	(0.056)	(0.061)
kidage	0.994***	0.965***	0.975***	2.141	1.958	2.036
	(0.317)	(0.317)	(0.315)	(2.136)	(2.135)	(2.124)
kidage_sq	−0.038***	−0.037***	−0.037***	−0.063	−0.057	−0.060
	(0.013)	(0.013)	(0.013)	(0.085)	(0.085)	(0.085)
kidgender	0.349***	0.332***	0.364***	2.410***	2.295***	2.341***
	(0.065)	(0.065)	(0.065)	(0.438)	(0.439)	(0.439)
kidbmi	0.004	0.003	−0.004	0.100*	0.094	0.099
	(0.009)	(0.009)	(0.009)	(0.059)	(0.060)	(0.060)
familysize		−0.050***	−0.011		−0.375***	−0.284**
		(0.019)	(0.020)		(0.127)	(0.133)
lnresivalue_tho		0.036**	0.038**		0.263**	0.116
		(0.018)	(0.019)		(0.122)	(0.125)
econ_status		0.047	0.053		0.003	0.075
		(0.032)	(0.033)		(0.218)	(0.220)
urban			0.256***			2.146***
			(0.073)			(0.495)
cohort_group	0.025	0.024	0.016	0.022	0.005	−0.005
	(0.023)	(0.023)	(0.023)	(0.157)	(0.157)	(0.157)
Province	control	control	control	control	control	control
Stochastic effect						
_all: Identity var.(R.cohort_group)	0.000	0.000	0.000	0.756	0.634	0.647
	(0.000)	(0.000)	(0.000)	(0.765)	(0.685)	(0.712)
period: Identity var.(_cons)	1.280	1.292	1.324	123.538	124.904	125.941
	(0.914)	(0.922)	(0.944)	(87.733)	(88.698)	(89.431)
LR test vs. linear model: chi2(2)	580.46***	587.56***	598.70***	1204.48***	1216.51***	1228.50***
Constant	−54.438	−52.629	−34.493	−58.788	−23.043	−1.696
	(46.261)	(46.238)	(46.118)	(317.408)	(316.825)	(316.216)
Observations	7,234	7,234	7,234	7,234	7,234	7,234

Standard errors in parentheses, ***, ** and * represent significant at the 1, 5 and 10% levels, respectively.

## 5 Further analysis

### 5.1 Gender heterogeneity in children

In the fixed effects analysis reported in Table 6, there is a significant difference in the impact of family education focus on children’s sports participation between boys and girls. Specifically, family education focus has a significant positive effect on boys’ sports participation, while the effect on girls is not significant. This indicates that family education focus significantly promotes boys’ enthusiasm and participation in sports, possibly due to a higher emphasis on boys’ athletic abilities and healthy development within families. Consequently, boys, with the support of family education focus, are more likely to engage in physical activities. However, the lack of significant impact of family education focus on girls’ sports participation may reflect that, in some families, even though there is educational focus, the support for girls’ sports participation is relatively weaker, or the educational focus is more concentrated on academics and other areas. Therefore, while family education focus does have an overall impact on children’s sports participation, the significant effect is predominantly observed in the boys’ group.

**Table 6:** Regression results for gender heterogeneity in children.

Variables	kidsport_fr		kidsport_hr	
	Boy	Girl	Boy	Girl
	(1)	(2)	(3)	(4)
Fixed effect				
child_education_level	0.090**	0.061	0.738**	0.383
	(0.045)	(0.043)	(0.320)	(0.264)
Household Level	control	control	control	control
Child Level	control	control	control	control
Family Level	control	control	control	control
Regional Level	control	control	control	control
Stochastic effect				
_all: Identity var.(R.cohort_group)	0.000	0.000	0.204	0.875
	(0.000)	(0.000)	(0.573)	(1.015)
period: Identity var.(_cons)	1.044	1.507	160.121	80.713
	(0.757)	(1.080)	(114.107)	(57.629)
LR test vs. linear model: chi2(2)	197.94***	414.29***	663.45***	549.12***
Constant	1.405	2.866***	−8.048	−6.891
	(0.915)	(0.886)	(6.731)	(5.381)
Observations	4,007	3,513	4,007	3,513

Standard errors in parentheses, ***, ** and * represent significant at the 1, 5 and 10% levels, respectively.

**Figure 4:**
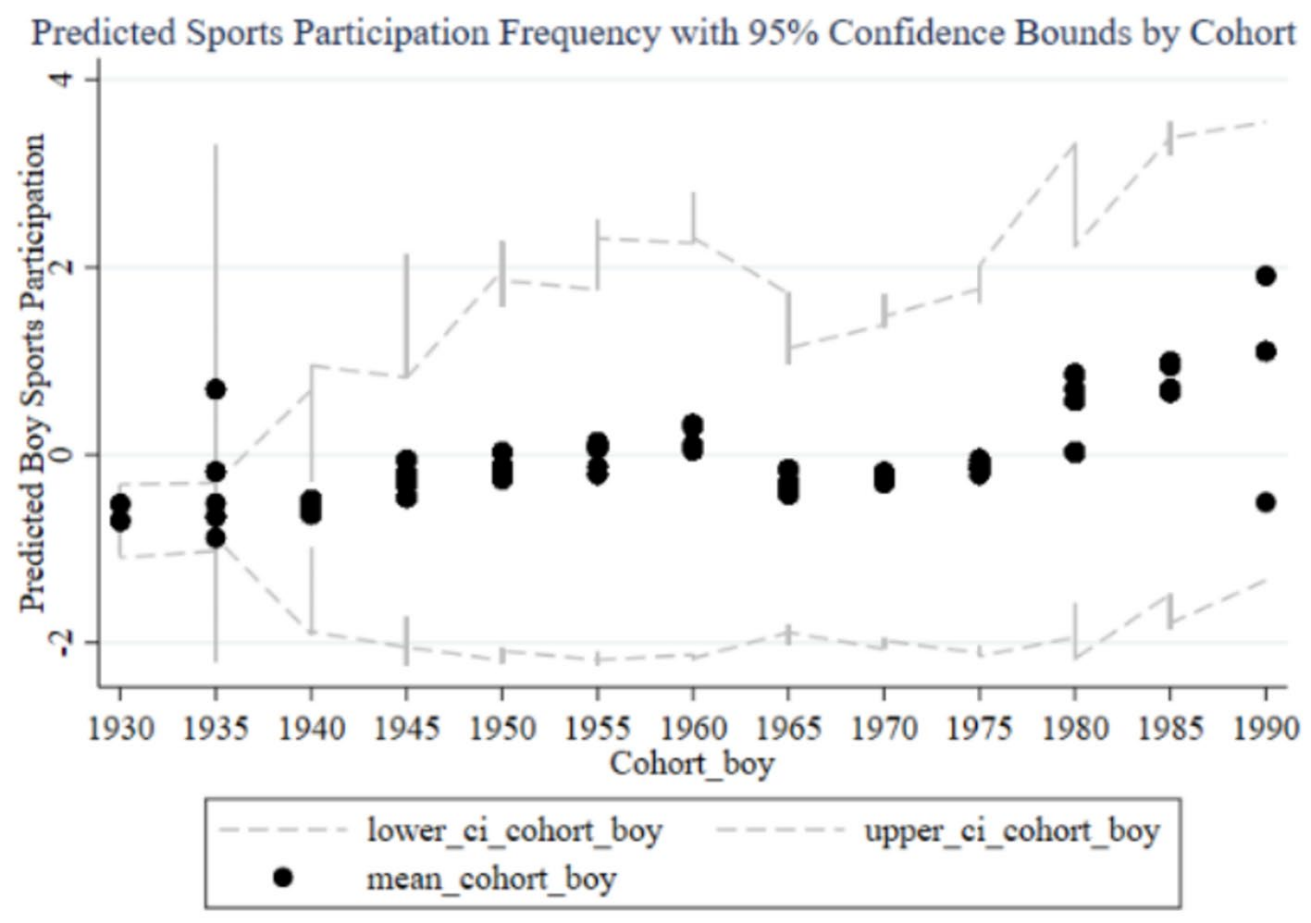
Cohort effects of family education focus on boys’ sports participation frequency.

**Figure 5:**
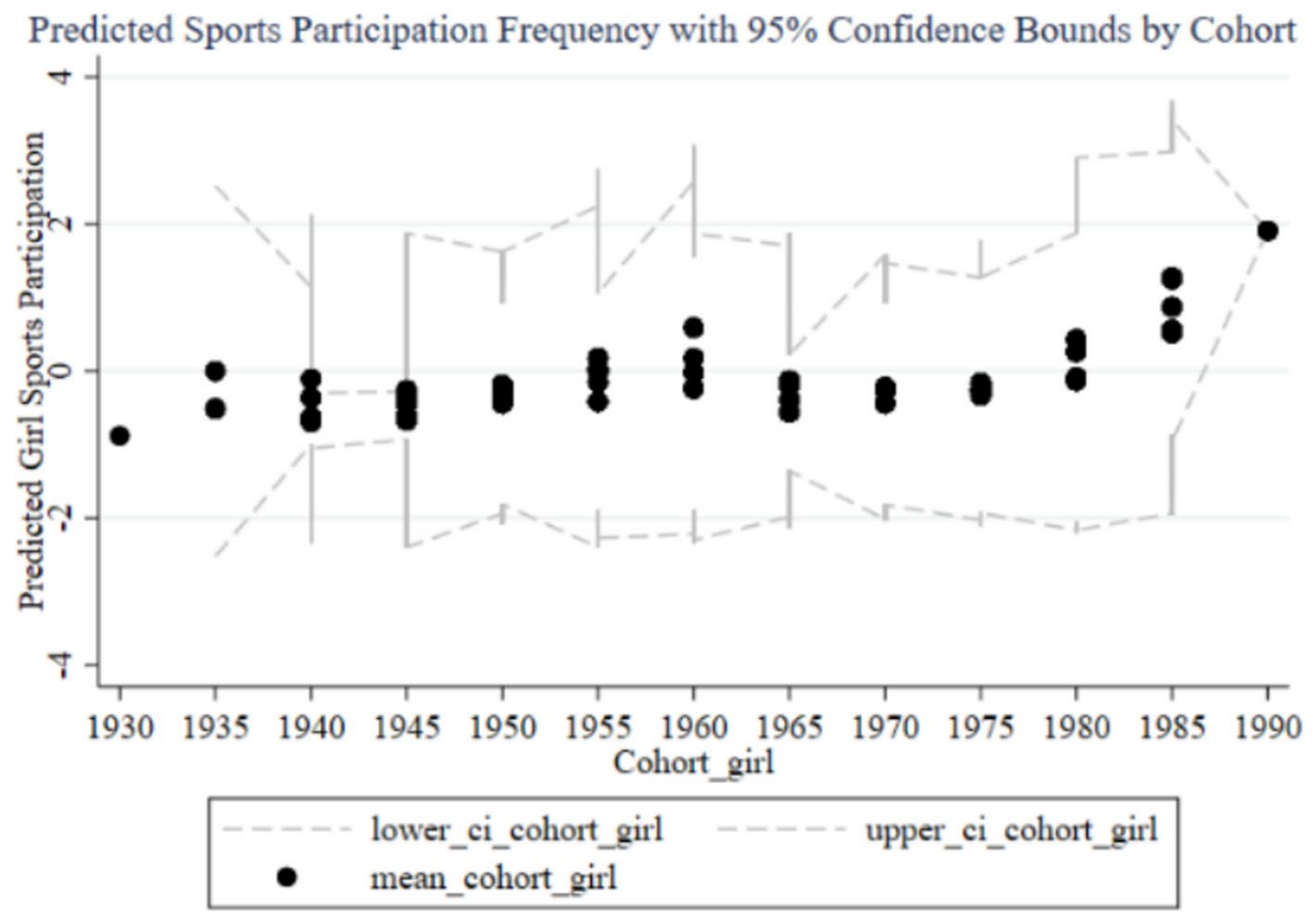
Cohort effects of family education focus on girls’ sports participation frequency.

**Figure 6:**
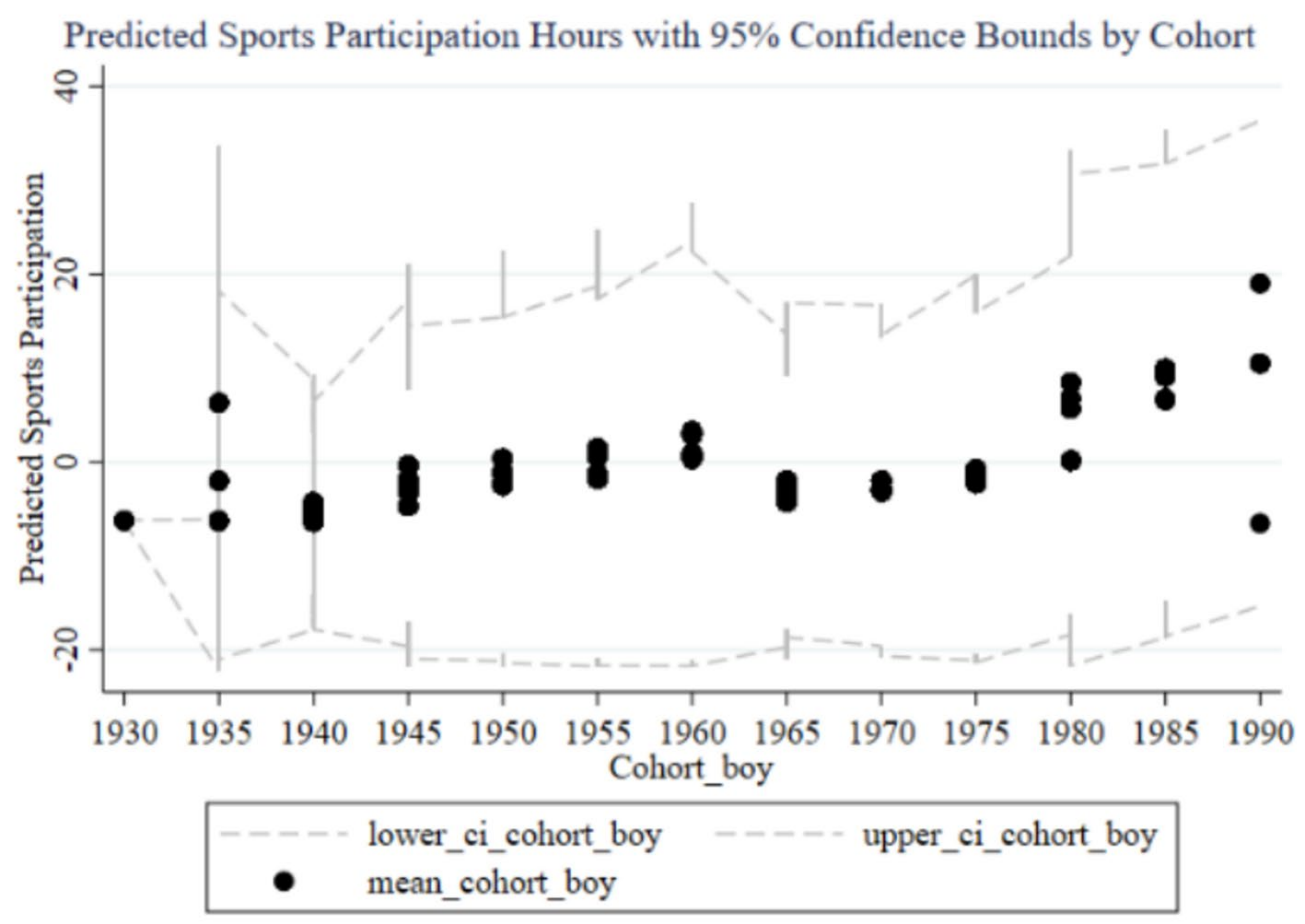
Cohort effects of family education focus on boys’ sports participation duration.

**Figure 7:**
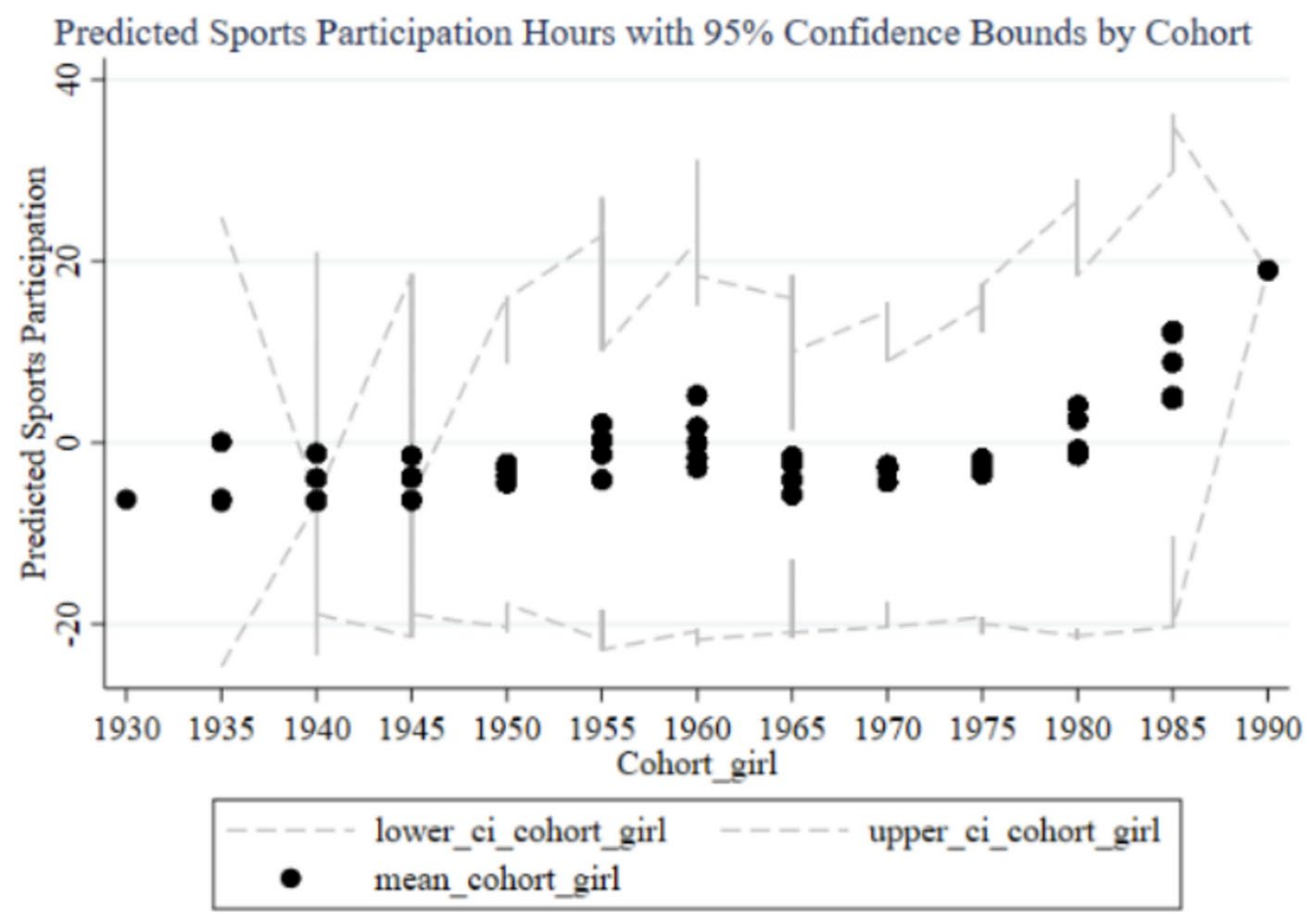
Cohort effects of family education focus on girls’ sports participation duration.

Figures 4–7 illustrate the impact of family education focus on the sports participation frequency and duration of boys and girls. Figure 4 shows a significant increase in boys’ sports participation frequency in recent cohorts, consistent with the positive effects found in the fixed effects analysis. The early negative effects (1930–1960) likely reflect societal restrictions, but the increasing family education focus has positively influenced boys’ participation over time, with narrow confidence intervals indicating stable effects. In contrast, Figure 5 reveals a less pronounced increase for girls, with wide confidence intervals suggesting lower estimation precision and an insignificant impact in the fixed effects analysis, reflecting weaker family support for girls’ sports participation. Figure 6 demonstrates similar trends in boys’ sports duration, with early negative effects turning positive from 1960, peaking in the 1985–1989 and 1990–1994 cohorts, and narrow confidence intervals indicating stability. The strong positive impact is likely due to families emphasizing boys’ athletic development. Conversely, Figure 7 shows less significant improvements in girls’ sports duration, with wide confidence intervals and lower estimation precision, indicating a need for greater family and social support to enhance girls’ sports participation. The fixed effects analysis confirms these findings, highlighting the disparity in family education focus between boys and girls. These findings reflect not only economic or structural inequalities but also deep-seated cultural perceptions in Chinese families, where boys’ physical development is often prioritized due to enduring gender stereotypes about strength, competition, and future roles.

### 5.2 Heterogeneity in family structure

The fixed effects analysis in Table 7 shows that family education focus has a significant positive impact on the sports participation frequency of children in two-parent families, whereas the impact on children in single-parent families is not significant. This indicates that in two-parent families, parental focus on education can significantly increase children’s sports participation frequency and duration. Two-parent families usually have more resources and time to better support and encourage children’s involvement in sports activities, thus the positive impact of family education focus on children’s sports participation frequency is significant. In contrast, the impact of family education focus on the sports participation frequency of children in single-parent families is not significant. This suggests that although there is educational focus in single-parent families, it does not significantly increase children’s sports participation frequency. The smaller sample size of single-parent families may contribute to the instability and uncertainty of the estimation results, increasing the estimation bias due to insufficient sample size. Single-parent families often face more resource and time constraints, and single parents, while managing family and work, may find it difficult to devote enough time and energy to focus on and support their children’s sports activities.

**Table 7:** Regression results for heterogeneity in family structure.

Variables	kidsport_fr		kidsport_hr	
	two_parent	single_parent	two_parent	single_parent
	(1)	(2)	(3)	(4)
Fixed effect				
child_education_level	0.079**	0.111	0.597***	0.189
	(0.033)	(0.112)	(0.226)	(0.554)
Household Level	control	control	control	control
Child Level	control	control	control	control
Family Level	control	control	control	control
Regional Level	control	control	control	control
Stochastic effect				
_all: Identity var.(R.cohort_group)	0.000	0.008	0.468	0.000
	(0.000)	(0.083)	(0.688)	(0.000)
period: Identity var.(_cons)	1.235	1.244	123.005	70.418
	(0.882)	(0.980)	(87.394)	(52.892)
LR test vs. linear model: chi2(2)	524.14***	39.56***	1122.18***	77.04***
Constant	−26.891	−247.026	−232.764	323.480
	(47.794)	(169.021)	(333.302)	(863.148)
Observations	6,680	554	6,680	554

Standard errors in parentheses, ***, ** and * represent significant at the 1, 5 and 10% levels, respectively.

**Figure 8:**
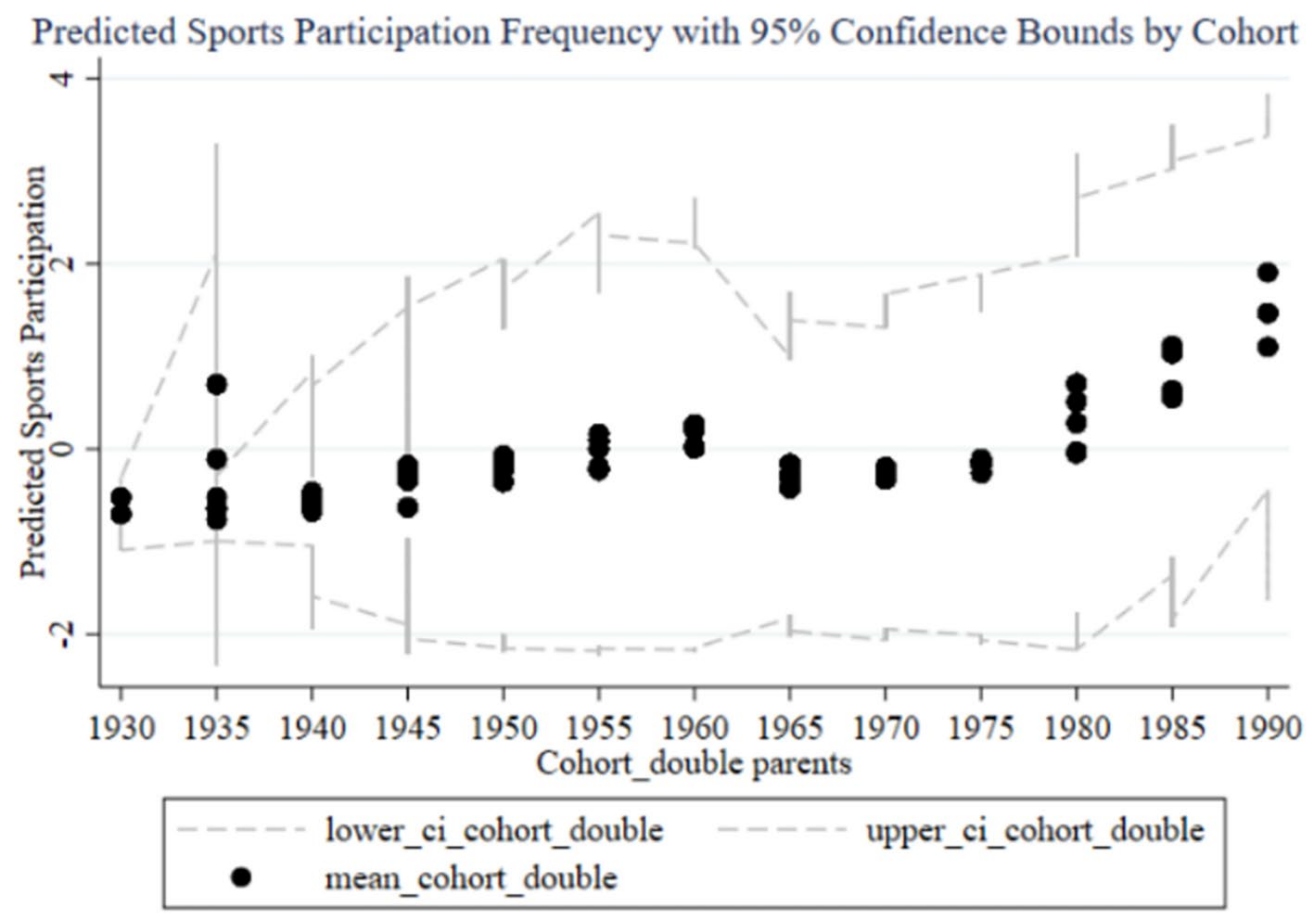
Cohort effects of family education focus on children’s sports participation frequency in two-parent families.

**Figure 9:**
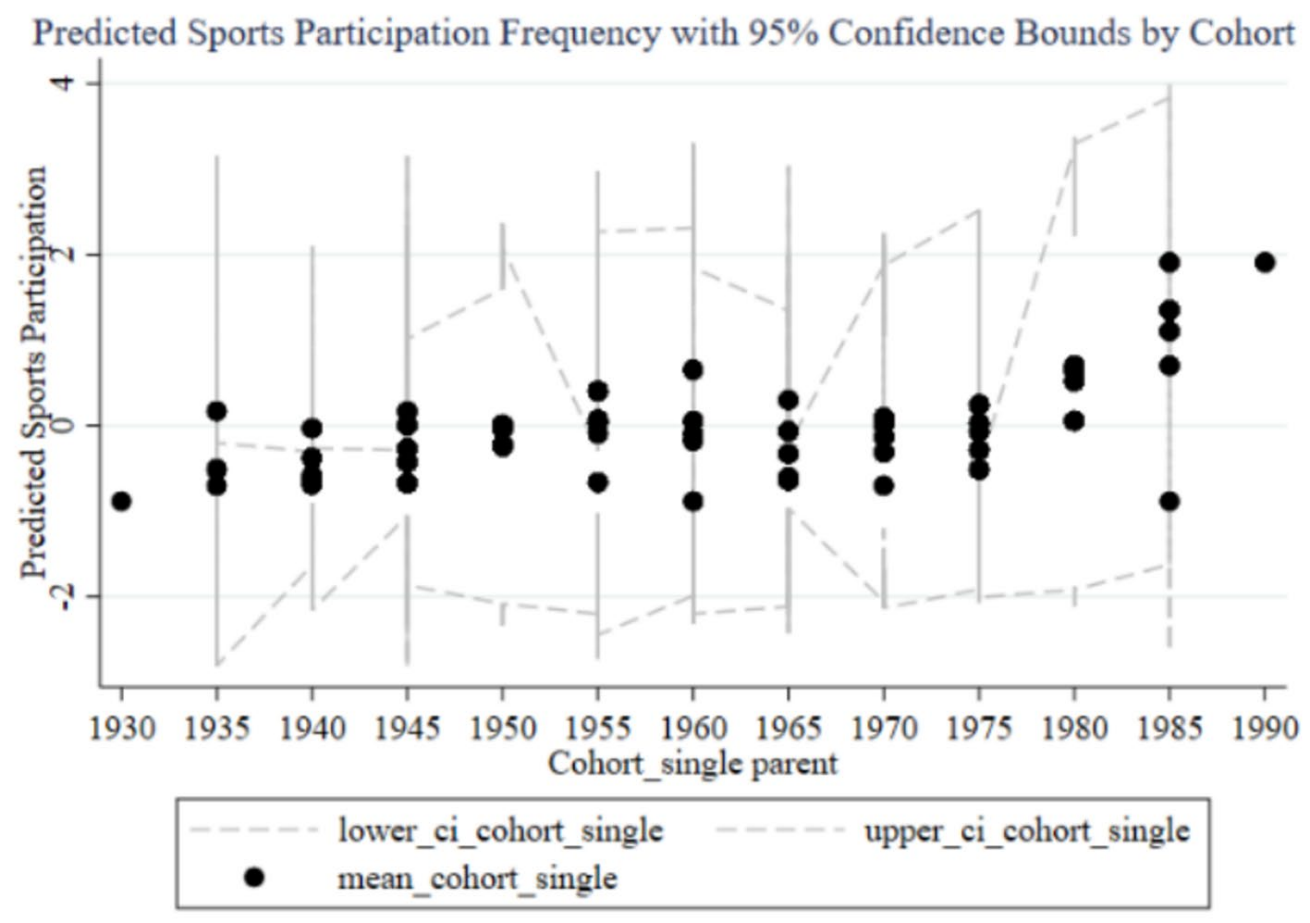
Cohort effects of family education focus on children’s sports participation frequency in single-parent families.

**Figure 10:**
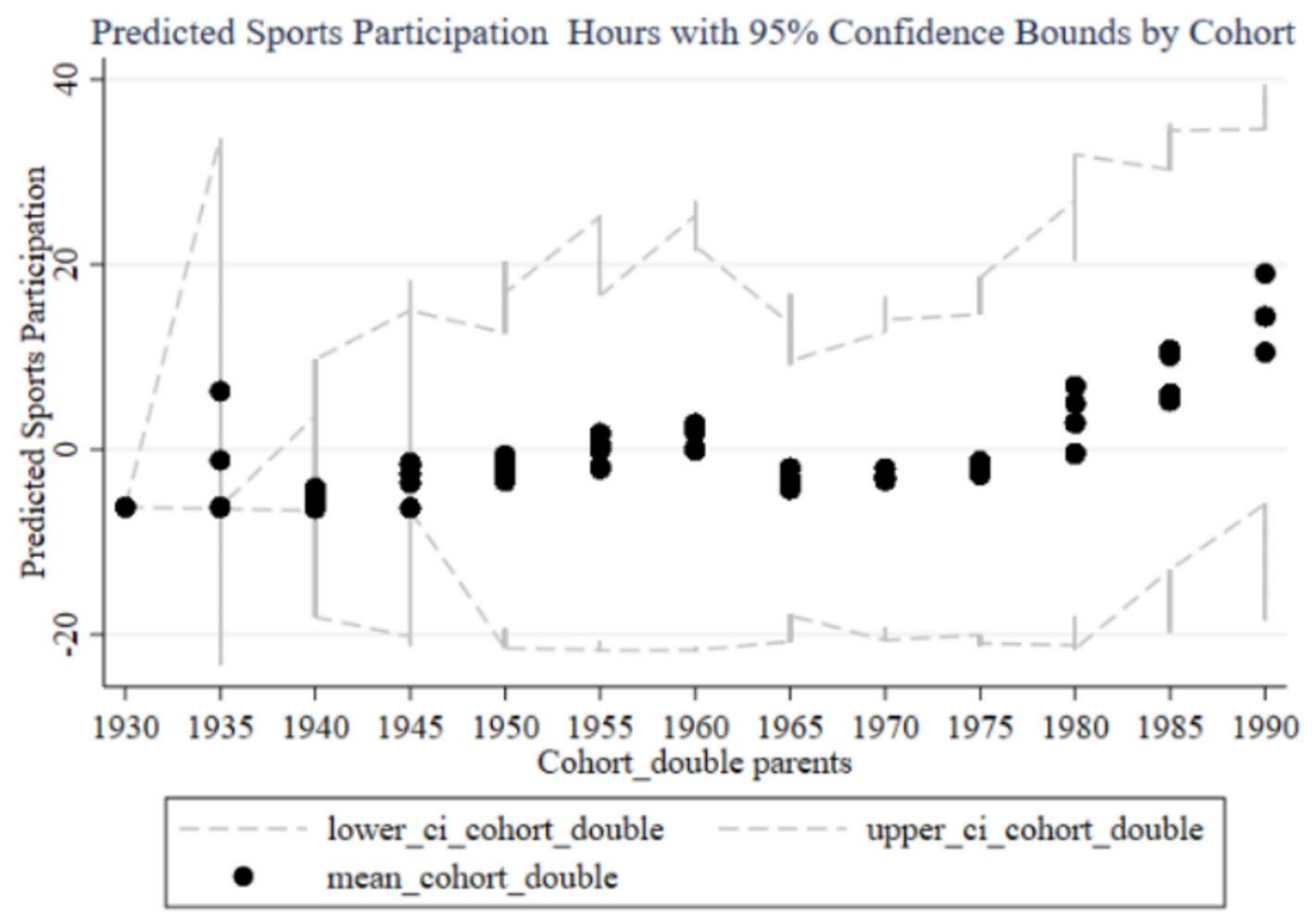
Cohort effects of family education focus on children’s sports participation duration in two-parent families.

**Figure 11:**
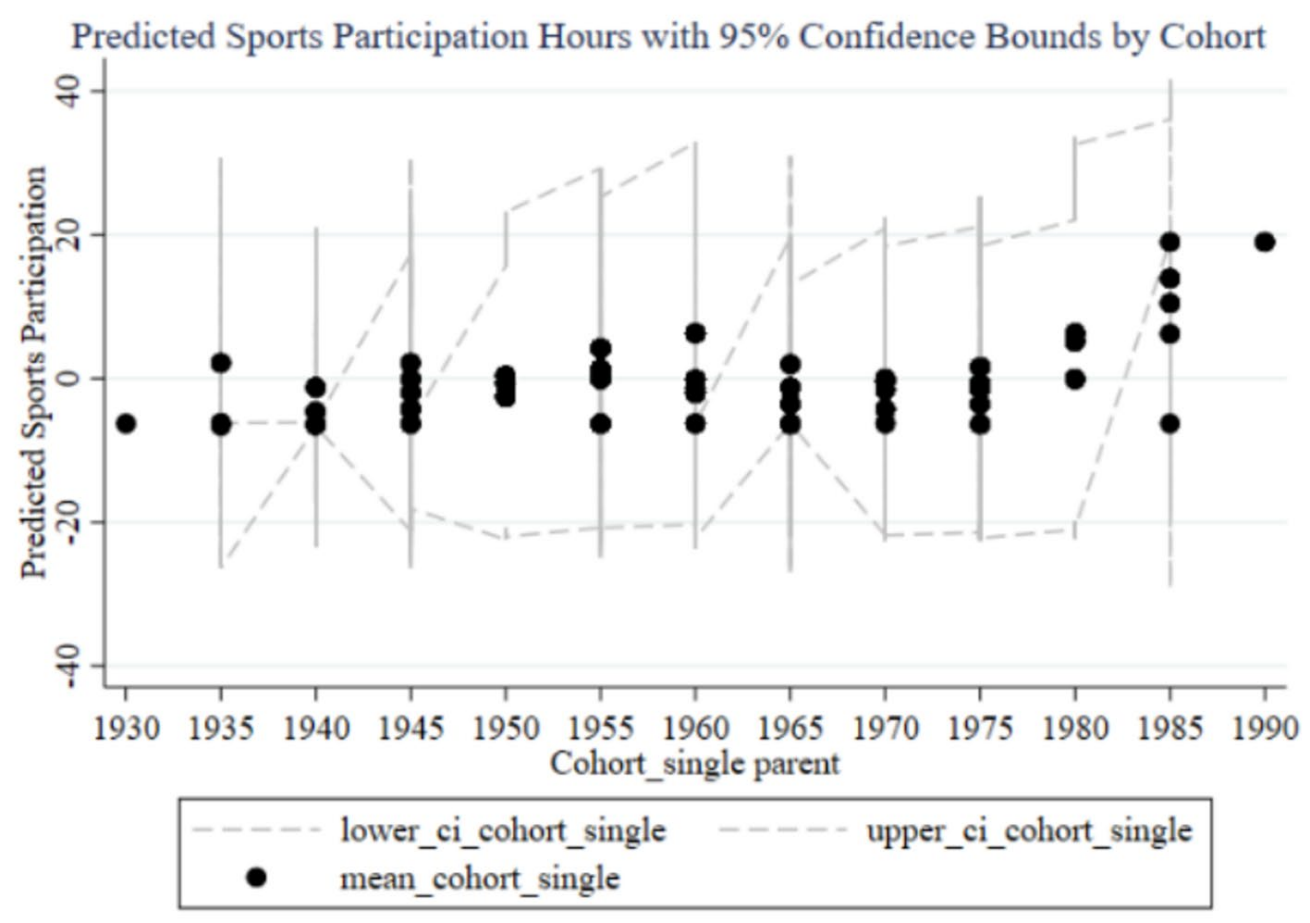
Cohort effects of family education focus on children’s sports participation duration in single-parent families.

Figures 8–11 illustrate the cohort effects of family education focus on the sports participation frequency and duration of children in two-parent and single-parent families. In two-parent families (Figures 8, 10), early cohort effects (1930–1960) are negative, indicating lower sports participation frequency and duration for children. From 1960 onward, these effects turn positive, peaking in the 1985–1989 and 1990–1994 cohorts, with narrow confidence intervals indicating high estimation precision and stable impact due to resource and time advantages. In single-parent families (Figures 9, 11), early cohort effects are also negative but with a smaller magnitude. Starting from 1980, the effects become positive, peaking in the 1990–1994 cohort. However, wider confidence intervals, especially during 1980–1990, suggest lower estimation precision and greater variability in the impact of family education focus. Overall, family education focus significantly positively impacts sports participation frequency and duration in two-parent families, while in single-parent families, the effects are less stable and significant, likely due to relative resource and time disadvantages.

### 5.3 Geographic heterogeneity

Table 8 shows the fixed effects of family education focus on children’s sports participation in urban and rural families. In urban areas, family education focus does not have a significant impact on children’s sports participation frequency. However, in rural areas, family education focus has a significantly positive impact on children’s sports participation frequency. Urban children may have more alternative activities (such as tutoring and extracurricular activities), which might reduce their time spent on sports. Conversely, rural families have relatively limited educational resources, so family education focus can significantly enhance children’s sports participation. In urban areas, the impact of family education focus on children’s sports participation duration is significantly positive at the 5% level, while in rural areas, the impact on sports participation freq3uency is significantly positive at the 10% level. This indicates that family education focus has a stronger influence on the duration of children’s sports participation in urban families.

**Table 8:** Geographic heterogeneity regression results.

Variables	kidsport_fr		kidsport_hr	
	Urban	Rural	Urban	Rural
	(1)	(2)	(3)	(4)
Fixed effect				
child_education_level	0.024	0.095**	0.822**	0.470*
	(0.053)	(0.039)	(0.390)	(0.244)
Household Level	control	control	control	control
Child Level	control	control	control	control
Family Level	control	control	control	control
Regional Level	control	control	control	control
Stochastic effect				
_all: Identity var.(R.cohort_group)	0.000	0.000	0.915	0.215
	(0.000)	(0.000)	(0.930)	(0.692)
period: Identity var.(_cons)	1.822	0.684	90.690	164.645
	(1.303)	(0.503)	(64.704)	(117.455)
LR test vs. linear model: chi2(2)	468.11***	131.87***	602.80***	580.42***
Constant	−77.395	−1.973	−126.658	81.477
	(70.300)	(60.419)	(522.820)	(385.787)
Observations	3,030	4,204	3,030	4,204

Standard errors in parentheses, ***, ** and * represent significant at the 1, 5 and 10% levels, respectively.

**Figure 12:**
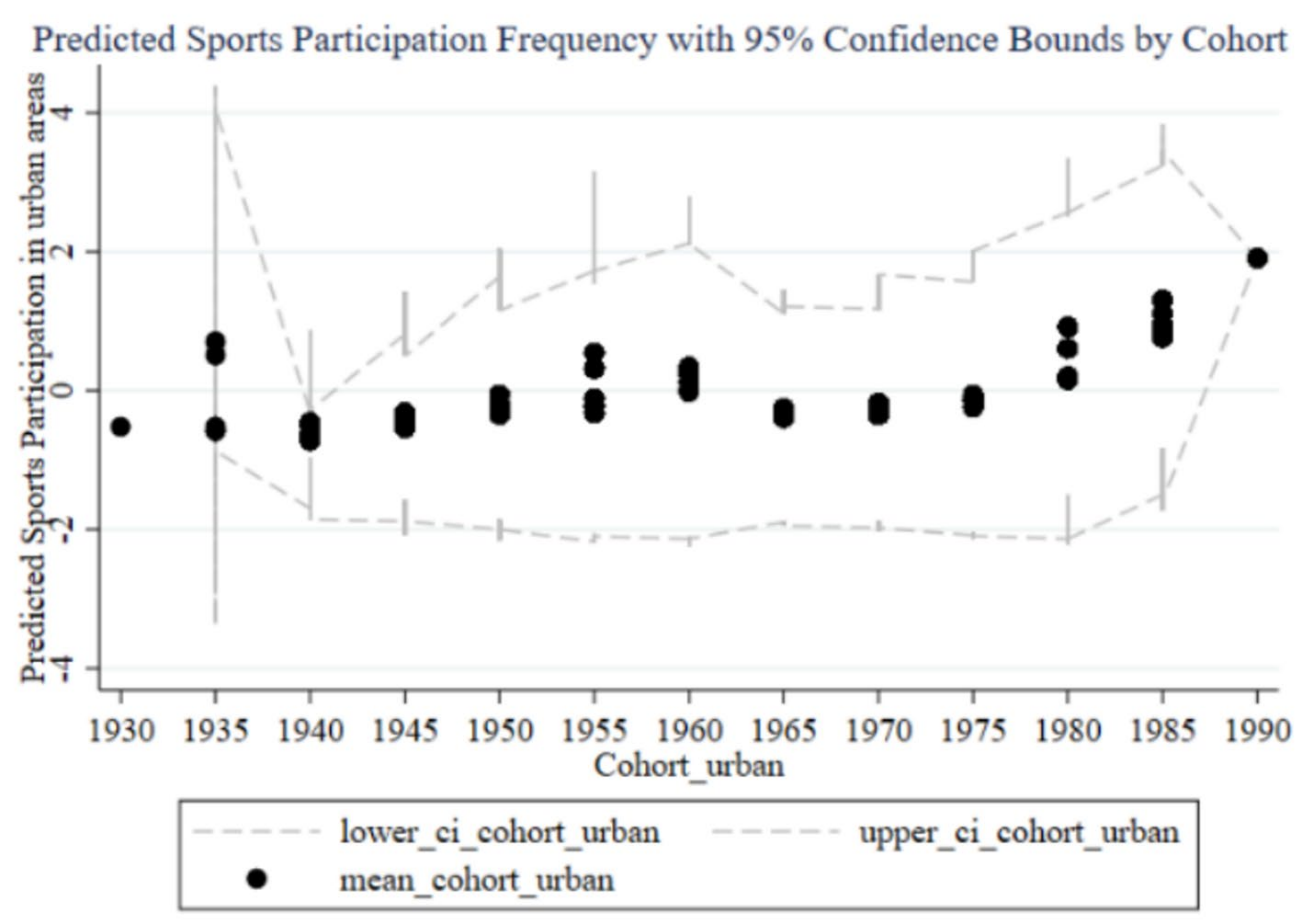
Cohort effects of family education focus on children’s sports participation frequency in urban families.

**Figure 13:**
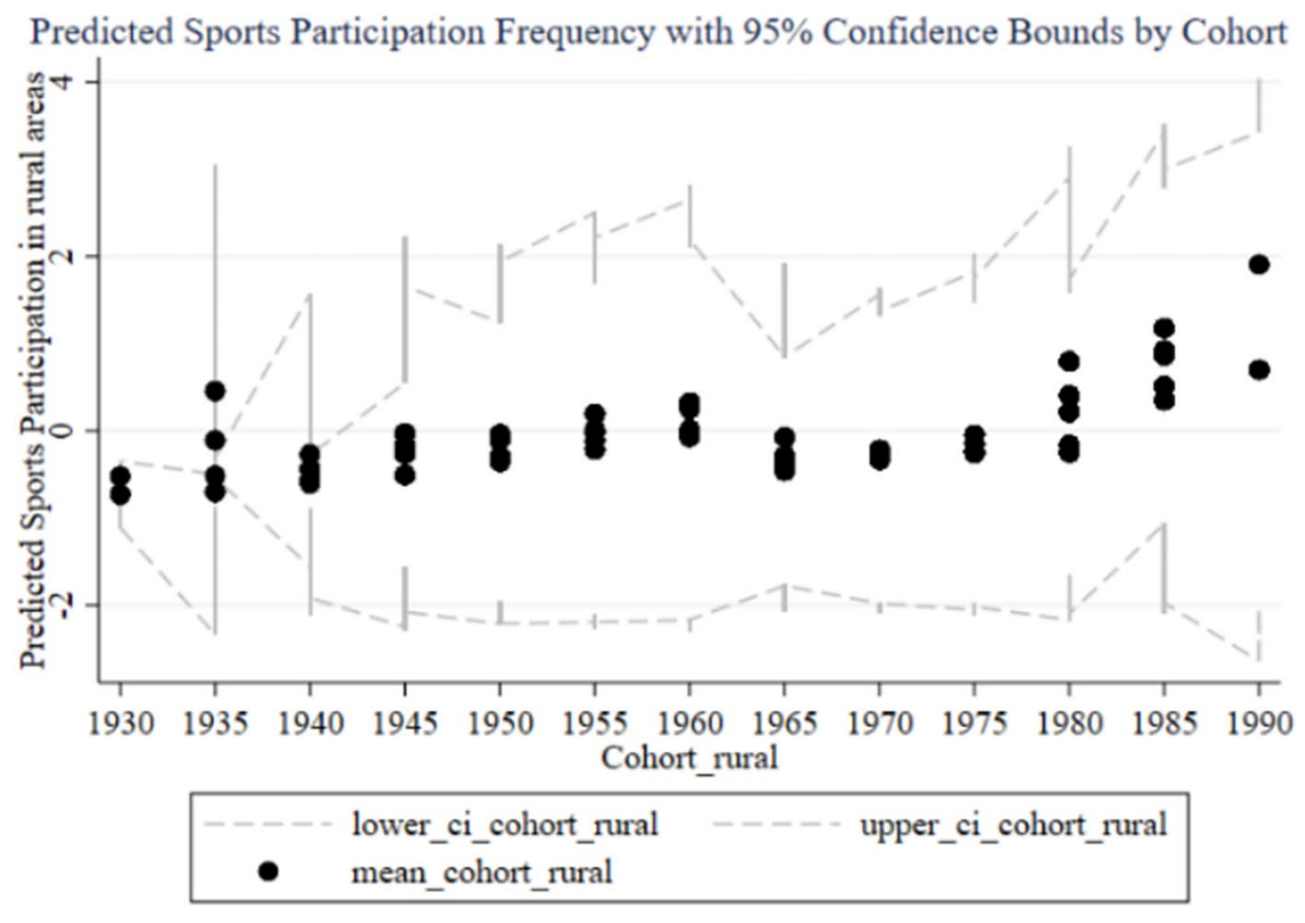
Cohort effects of family education focus on children’s sports participation frequency in rural families.

**Figure 14:**
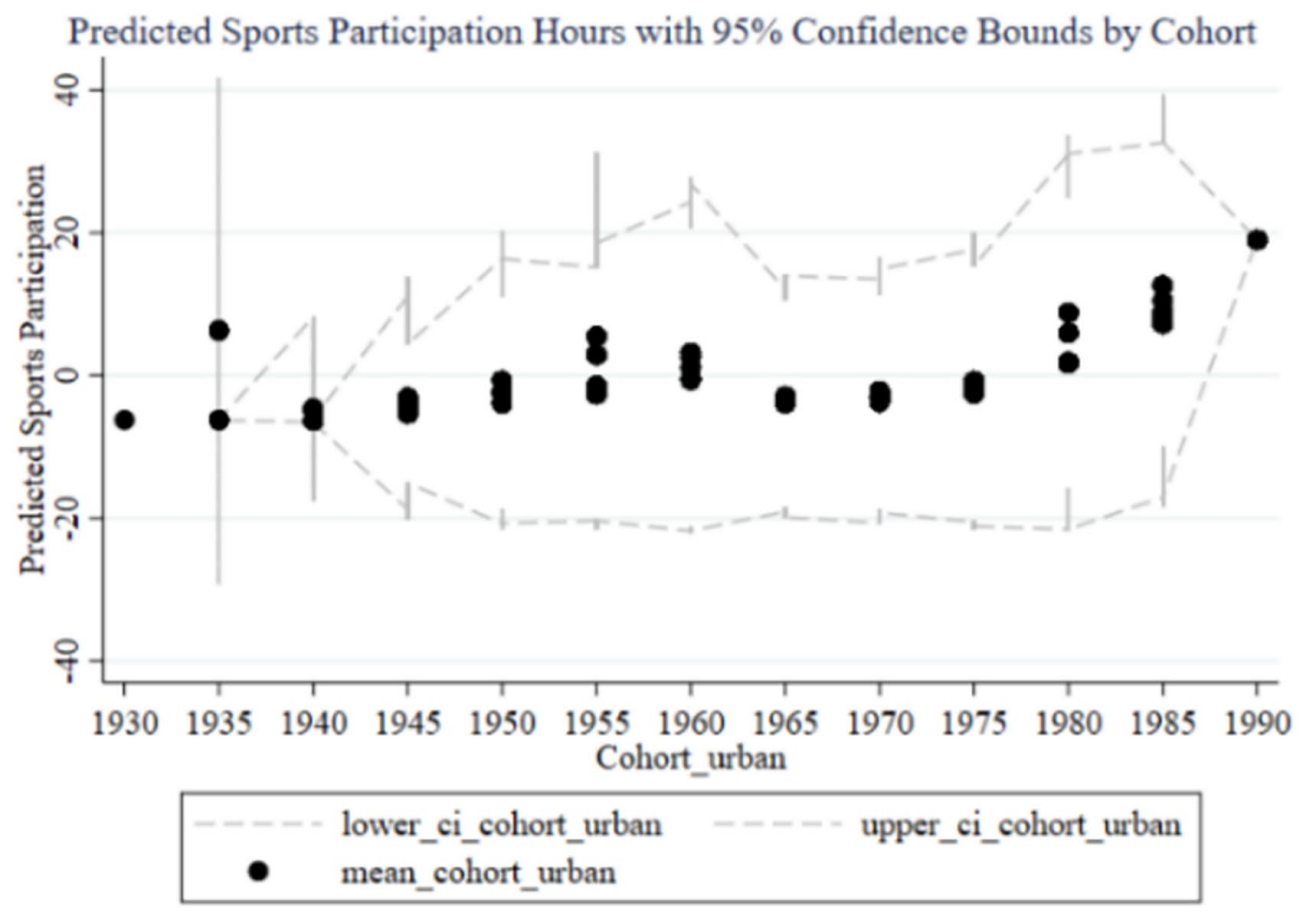
Cohort Effects of family education focus on children’s sports participation duration in urban families.

**Figure 15:**
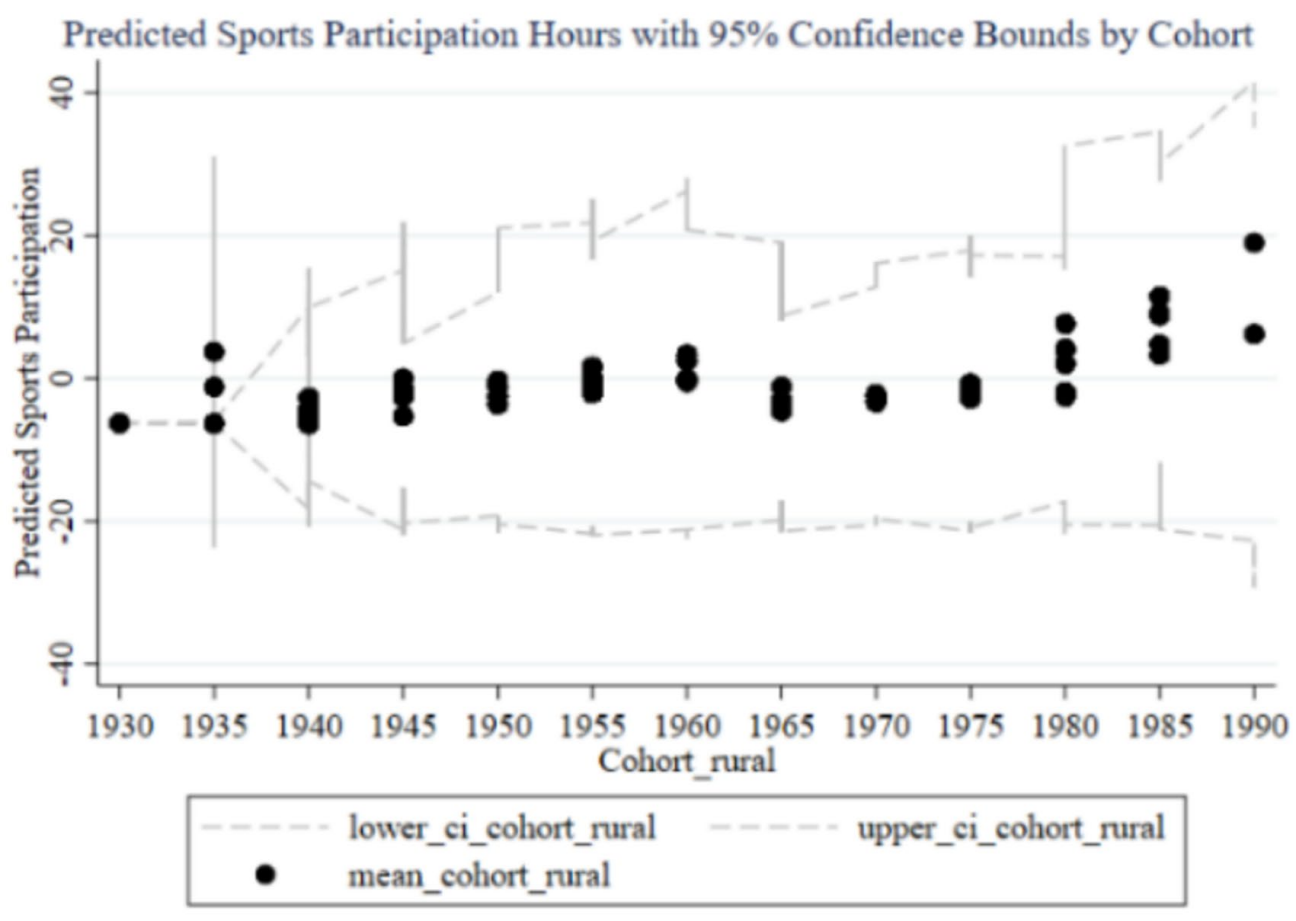
Cohort effects of family education focus on children’s sports participation duration in rural families.

Figures 12–15 illustrate the cohort effects of family education focus on the sports participation frequency and duration of children in urban and rural families, respectively. In urban families, early cohort effects (1930–1960) are negative for both frequency (Figure 12) and duration (Figure 14), indicating relatively lower sports participation for children in these cohorts. From 1960 onward, the cohort effects turn positive, peaking in the 1985–1989 and 1990–1994 cohorts, with narrow confidence intervals suggesting high estimation precision and a stable positive impact due to resource and time advantages. In rural families, early cohort effects (1930–1960) are also negative with a small magnitude for both frequency (Figure 13) and duration (Figure 15). Starting from 1980, the effects gradually turn positive, peaking in the 1990–1994 cohort. However, the wider confidence intervals, especially during 1980–1990, indicate lower estimation precision and a more variable impact of family education focus, likely due to relative disadvantages in resources and time.

## 6 Conclusions and policy implications

This study examined the temporal evolution of the relationship between family education focus and children’s sports participation using data from the CFPS (2014–2020) and a Hierarchical Age-Period-Cohort (HAPC) model. The analysis reveals that higher parental educational focus is positively associated with children’s frequency and duration of sports participation. Notably, this effect varies by cohort: parents born after 1980 exhibit a significant positive influence, marking a generational shift from a narrow academic emphasis toward more holistic parenting styles that prioritize children’s physical development. This reflects evolving psychological values regarding child well-being, health, and balance in education.

These findings align with and can be interpreted through several well-established psychological frameworks. Social Learning Theory explains how children internalize parental behaviors and priorities, such as valuing or neglecting physical activity. When parents actively engage in sports or demonstrate consistent attention to their children’s physical development, they model behaviors that children are likely to adopt. Self-Determination Theory (SDT) provides insight into how autonomy-supportive parenting—characterized by encouragement, structure, and emotional warmth—fosters intrinsic motivation for physical activity in children. Lastly, Ecological Systems Theory emphasizes the importance of the family as a foundational microsystem. Within this environment, parental norms and expectations set the tone for a child’s behavioral development and health-related values.

From a psychological policy standpoint, interventions should go beyond infrastructure investment to reshape parental attitudes and behaviors. This could include parent-targeted psychoeducation programs, workshops on positive sports modeling, and community campaigns to normalize physical activity as a family value.

Based on the evidence, we propose four targeted policy recommendations:

*First*, policies should prioritize enhancing parents’ psychological understanding and behavioral modeling regarding children’s sports development. Given that higher family education focus correlates with increased sports participation, governments and schools can implement structured *parent education programs* that emphasize the long-term cognitive, emotional, and social benefits of physical activity. These programs should draw on social learning principles to improve parents’ role-modeling capacity and reshape the perception that sports are secondary to academic achievement.

*Second*, in light of the generational shift identified among parents born after 1980, policies should *leverage this cohort as a driver of cultural change*. These parents can serve as “peer educators” or community ambassadors in promoting holistic child development. Government-supported initiatives can train interested 80s-generation parents to lead workshops, share parenting strategies, and participate in community outreach campaigns that normalize and celebrate active lifestyles within families.

*Third*, to address group-specific disparities—particularly for girls, rural children, and those from single-parent families—*targeted interventions must go beyond infrastructure to reshape attitudes and access*. In the case of girls, efforts should confront traditional cultural beliefs that devalue female participation in sports. This includes media representation, school programming that challenges gender stereotypes, and parental workshops that normalize sports as a beneficial pursuit for all children, regardless of gender.

*Finally*, sustainable improvements require an *ecosystem-level integration* of family, school, and community roles. Policies should encourage local governments and education bureaus to co-develop “Family-Engaged Physical Literacy Plans” that coordinate physical education curricula, after-school offerings, and weekend family events. This alignment can foster children’s motivation through autonomy, competence, and relatedness—thereby turning short-term participation into a lifelong habit.

## Data Availability

The datasets presented in this study can be found in online repositories. The names of the repository/repositories and accession number(s) can be found at: https://www.isss.pku.edu.cn/cfps/sjzx/gksj/index.htm.
